# Molecularly Imprinted Nanoparticles Based Sensor for Cocaine Detection

**DOI:** 10.3390/bios10030022

**Published:** 2020-03-04

**Authors:** Roberta D’Aurelio, Iva Chianella, Jack A. Goode, Ibtisam E. Tothill

**Affiliations:** 1 Advanced Diagnostics and Sensors Group, Cranfield University, Cranfield, Bedfordshire MK43 0AL, UK; i.tothill@cranfield.ac.uk; 2 School of Biomedical Sciences, Faculty of Biological Sciences, University of Leeds, Leeds LS2 9JT, UK; jack.goode@gmail.com

**Keywords:** cocaine, drugs of abuse, electrochemical impedance spectroscopy, molecularly imprinted polymer nanoparticles, EIS sensor

## Abstract

The development of a sensor based on molecularly imprinted polymer nanoparticles (nanoMIPs) and electrochemical impedance spectroscopy (EIS) for the detection of trace levels of cocaine is described in this paper. NanoMIPs for cocaine detection, synthesized using a solid phase, were applied as the sensing element. The nanoMIPs were first characterized by Transmission Electron Microscopy (TEM) and Dynamic Light Scattering and found to be ~148.35 ± 24.69 nm in size, using TEM. The nanoMIPs were then covalently attached to gold screen-printed electrodes and a cocaine direct binding assay was developed and optimized, using EIS as the sensing principle. EIS was recorded at a potential of 0.12 V over the frequency range from 0.1 Hz to 50 kHz, with a modulation voltage of 10 mV. The nanoMIPs sensor was able to detect cocaine in a linear range between 100 pg mL^−1^ and 50 ng mL^−1^ (R^2^ = 0.984; *p*-value = 0.00001) and with a limit of detection of 0.24 ng mL^−1^ (0.70 nM). The sensor showed no cross-reactivity toward morphine and a negligible response toward levamisole after optimizing the sensor surface blocking and assay conditions. The developed sensor has the potential to offer a highly sensitive, portable and cost-effective method for cocaine detection.

## 1. Introduction

Cocaine is classified as a central nervous system stimulant and is the most abused drug in the world, after cannabis. With over 655 tons detained yearly, cocaine is also one of the most seized illicit drugs worldwide [1] and is one of the major recreational drugs illegally trafficked in European countries, where there are more than 17.5 million users [2]. Illicit-drugs-related crimes are of huge concern due to the burden on law enforcement agencies (LEAs) and healthcare systems, with associated social and economic problems.

To detect, control and manage drugs of abuse trafficking, LEAs make use of the powerful olfactory system of “sniffer dogs”. After an appropriate training, dogs are able to “smell and detect” very low concentrations (in the range of ppb) of illicit drugs concealed in various items, including packages and containers [3,4]. However, sniffer dogs can be prone to work fatigue and can be deceived by handlers [5]. Forensic chemistry can provide evidence on whether a suspected substance contains illegal drugs by means of reproducible scientific methods, thus providing unequivocal evidence of the drug-related offence [6]. Generally, suspected samples (either biological or environmental) are analyzed by presumptive and confirmatory tests. The former comes as rapid detection kits or devices, which are mainly screening tests indicating whether illegal drugs may be present or not [7]. Currently, onsite screening methods rely on Ion Mobility Spectroscopy (IMS) [8], competitive inhibition immunoassay [9] and colorimetric tests [7]. Nevertheless, these methods provide only qualitative or semi-quantitative results; they are prone to false-positive and negative results and many require trained personnel for operation. Confirmatory analyses are currently performed in accredited ISO 17025 laboratories, through several complex procedures and expensive analytical tools, such as GC–MS and LC–MS [10].

Therefore, there is a need for new technologies that can provide fast screening tests with a high level of sensitivity and specificity. These technologies may speed up the investigative activities, thus reducing the false-positive/false-negative rate and, hence, confining the demand of confirmatory tests only on truly positive samples. Hence, the aim of this work was to develop a rapid and specific sensor able to detect cocaine at trace levels, to be used in diverse onsite testing scenarios, to tackle cocaine trafficking. While it is difficult to reproduce the complexity of the olfactory systems, electrochemical biosensors can offer an analogous way to transform the binding of the analyte to its sensing receptor into an electrical signal. Examples of electrochemical sensors for cocaine detection using aptamers or biomolecules have already been described in the literature [11,12,13]. Among all the electrochemical techniques available, in recent years, electrochemical impedance spectroscopy (EIS) has gained attention due to its ability of detecting the target molecules at very low concentrations. Compared to amperometry and potentiometry, EIS is able to detect minimal changes at the sensor surface boundaries, thus leading to several advantages, such as a wide linear range, low limits of detection and direct assay mode. The method can also preserve the sample for further confirmatory analysis [14,15]. The basic principle behind EIS is the electrical impedance, which indicates the resistance that an electrical circuit presents to the flow of an alternating current (AC), generated by applying a small alternating voltage (AV) [16]. EIS can be performed with or without a redox probe (redox couple), such as potassium ferricyanide/ferrocyanide ([Fe(CN)_6_]^3−/4−^), added to the solution. When the redox probe is present, faradic current is gathered, thus leading to faradic EIS sensor. In this case, the resistance charge transfer (Rct) electrical element is usually affected by the events occurring at the electrode surface, such as the binding between a sensing receptor and its target [17].

In order to obtain a sensor suitable for onsite testing, it is highly desirable to employ stable and robust sensing elements. Previous works have demonstrated that the use of molecularly imprinted polymers in electrochemical sensing can result in robust, sensitive and specific diagnostic systems [18,19,20]. Particularly, molecularly imprinted nanoparticles (nanoMIPs), prepared using a solid phase, have shown to be a powerful and robust mimic of antibodies in sensors and assays [21,22,23], while providing convenient and animal-free synthesis. NanoMIPs have shown to be stable at a wide range of temperature and to possess a long shelf-life, with no need of refrigeration and preservation [22,24]. As such, nanoMIPs are the ideal receptor candidate for sensors that have to operate in unpredictable environmental conditions, which might contain denaturing agents and degrading enzymes capable of denaturing bio-derived receptors (protein, antibodies and aptamers).

Therefore, nanoMIPs and EIS were applied in this work for the first time to develop a highly sensitive and specific affinity sensor to detect traces of cocaine. The resulting nanoMIPs EIS sensor was able to detect cocaine in the low nM range, demonstrating potential application as a cheap and portable analytical tool to use in investigative activities of illicit drugs trafficking.

## 2. Materials and Methods

### 2.1. Reagents

Cocaine nanoMIPs in acetonitrile were provided by Professor Piletsky’s group (University of Leicester) [25]. During the synthesis, nanoMIPs were functionalized with primary amino groups, thus allowing their covalent attachment to the sensor surface. Cocaine hydrochloride, morphine hydrochloride (trihydrate) and levamisole were purchased from Sigma Aldrich (Dorset, UK) and were handled according to the Home Office (UK) guidelines. The 3-(N-Morpholino) propanesulfonic acid powder (MOPS) was purchased from Sigma-Aldrich (Dorset, UK) and used to make buffer solutions. The 11-mercaptodecanoic acid (MUDA) was purchased from Sigma-Aldrich (Dorset, UK) and was dissolved in 50 mL of ethanol (pure ethyl alcohol, anhydrous, ≥99.5%), at a concentration of 5 mM. N-Hydroxysuccinimide (NHS) and 1-Ethyl-3-(3-dimethylaminopropyl) carbodiimide (EDC) were purchased from Thermo Scientific (Rugby, UK) and dissolved in water, to obtain 0.1 and 0.4 M solutions, respectively. Ethanolamine (MEA), polyvinyl alcohol (PVA), Bovine Serum Albumin (BSA), milk proteins and Tween 20 were purchased from Sigma-Aldrich (Dorset, UK) and used as blocking agents. The 10 mM redox couple solution ([Fe(CN)_6_]^3−/4−^) was prepared by dissolving potassium ferrocyanide (K_4_[Fe(CN)_6_]) and potassium ferricyanide (K_3_[Fe(CN)_6_]) in MOPS (10 mM, pH 7.4). All the reagents were of analytical grade. All the aqueous solutions were prepared, using ultrapure water (18 MΩ·cm) that was filtered using 0.2 µm sterile filters.

### 2.2. Apparatus and Measurements

NanoMIPs’ diameter size was assessed by Transmission Electron Microscopy (TEM, CM20, Phillips, Amsterdam, The Netherlands) and Dynamic Light Scattering (DLS-Zetasizer Nano-S, Malvern Instruments Ltd, Malvern, UK). To perform the DLS analysis, the nanoMIPs solvent was exchanged to water via the Eppendorf concentrator 5301 (Eppendorf Ltd, Stevenage, UK) and was then filtered, using a 0.45 µm filter. In order to perform the TEM analysis, 10 µL of nanoMIPs solution was deposited on the TEM sample copper holder, and the solvent was allowed to evaporate.

The quality of the cocaine nanoMIPs attachment on the electrode surface was investigated by Atomic Force Microscopy (AFM, Dimension 3100, Bruker, Billerica, MA, USA). For AFM analysis, the electrodes were rinsed with doubled-deionized water and dried under a gentle stream of nitrogen. Furthermore, sensor surface characterization study was performed by recording EIS measurement at each functionalization step.

PalmSens4 (PalmSens BV, Houten, The Netherlands) was used as potentiostat/EIS analyzer. The sensor used in this work was DPR C 220AT (DropSens, Asturias, Spain), with a gold working electrode of 4 mm in diameter and was connected to the PalmSens4 via a universal sensor connector. The connector was coupled to the instrument by a double-shielded cable with 2 mm banana connectors. The PalmSens was connected to a PC, on which the dedicated PStrace 5 software (PalmSens BV, The Netherlands) was installed. A Faraday cage was used to perform all the electrochemical measurements. EIS was recorded at a 0.12 V potential from 0.1 Hz to 50 kHz, with a modulation voltage of 10 mV. All sensors surfaces were rinsed and dried before each step of the assay for the EIS measurements. All EIS measurements were performed in 10 mM redox couple solution ([Fe(CN)_6_]^3−/4−^) and at room temperature (23 ± 1 °C). All experimental data were collected and fitted onto the Randles equivalent circuit by PStrace 5.2 (PalmSens BV, The Netherlands).

### 2.3. NanoMIP Sensor Fabrication and Characterization

**Scheme 1:**
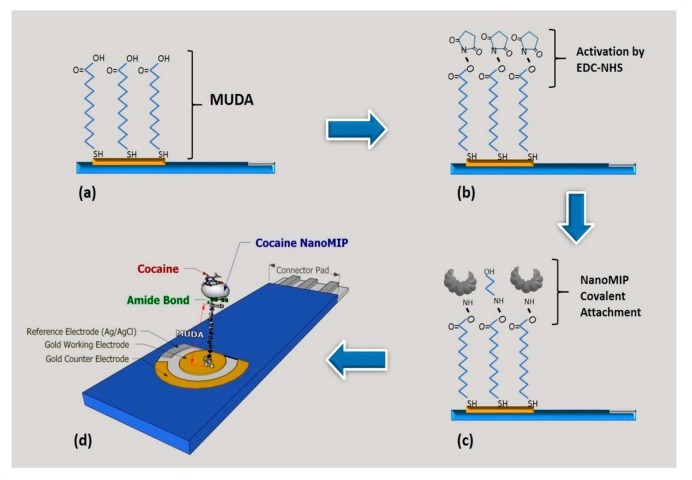
Steps of attaching the nanoMIPs onto gold working electrode surface (DPR C220AT, DropSens). (**a**) SAM formation; (**b**) carboxylic group activation by EDC-NHS; (**c**) nanoMIPs covalent attachment via amine coupling. (**d**) 3D scheme of the final nanoMIPs sensor for cocaine detection. Scheme not in scale.

Gold electrodes were first cleaned according to the manufacturer instructions and then incubated overnight in an ethanol solution containing 5 mM MUDA, to create a self-assembly monolayer (SAM). Afterward, the sensors were rinsed with deionized water and dried with nitrogen. A mixture of EDC/NHS was used to activate the carboxylic groups of MUDA, thus enabling the attachment of the nanoMIPs (5 mg mL^−1^) via amine coupling chemistry [26]. Cocaine nanoMIPs were left in contact with the activated surface for 1 h at 23 ± 1 °C. Then, the electrodes were again rinsed with water and dried under a gentle nitrogen stream. The unreacted activated carboxylic groups were capped with ethanolamine 1 M, pH 8.5. The general attachment protocol is displayed in Scheme 1.

### 2.4. Development and Optimization of the EIS NanoMIPs Affinity Sensor

#### 2.4.1. Blocking Agent Optimization

Several blocking agents were used in combination with ethanolamine (1 M, pH 8.5) to deactivate remaining reactive groups on the sensor surface after attachment of the MIPs: (1) ethanolamine with BSA (0.1%, w/v) and Tween 20 (1%, v/v), pH 6.0; (2) ethanolamine with milk protein (10%, v/v), pH 6.0; (3) ethanolamine with BSA (0.1%, w/v) and Tween 20 (1%, v/v), pH 7.4; and (4) ethanolamine with PVA (1%, v/v), pH 7.4. For each blocking agent, the average (±SD) of the sensor response was plotted and compared. The blocking agent with the Rct value closer to zero was chosen as the best and applied to assess the optimized sensor sensitivity.

#### 2.4.2. Cocaine Assay

The sensor ability to detect cocaine was tested by performing cumulative assays. Specifically, increasing concentrations of cocaine (100 pg mL^−1^–50 ng mL^−1^) were prepared in water and then in MOPS. Each concentration, from the lowest to the highest, was incubated on the sensor surface for 30 min, and the EIS readout was recorded. To confirm that the analyte was binding to the cocaine nanoMIPs, a cumulative assay was also carried out onto sensors fully functionalized but without nanoMIPs. All the experiments were carried out on independent sensors and at least in triplicates.

#### 2.4.3. Specificity Assays

Sensor specificity was evaluated against morphine, another commonly abused drug, and the cutting agent levamisole. Morphine and levamisole were dissolved in MOPS, and solutions of increasing concentrations (from 100 pg mL^−1^ to 50 ng mL^−1^) were prepared and used to perform cumulative assays. To assess the specificity, the sensor response was evaluated against the cocaine sensor response. All the experiments were carried out on independent sensors and at least in triplicates.

### 2.5. Data Processing and Analysis

The experimental data were fitted onto an appropriate equivalent circuit by an EIS spectrum analyser® v1.0, and the obtained Rct (Ω) value was expressed as a percentage of the blank signal (expressed as −Δ% Rct, when negative values were obtained), thus standardizing the sensor response across the sensors, as reported by Ahmed et al. [27]. Data were collected and analyzed by Microsoft® Excel® and IBM® SPSS® Statistics 24.0.

## 3. Results and Discussion

### 3.1. NanoMIPs Characterization Study

**Figure 1:**
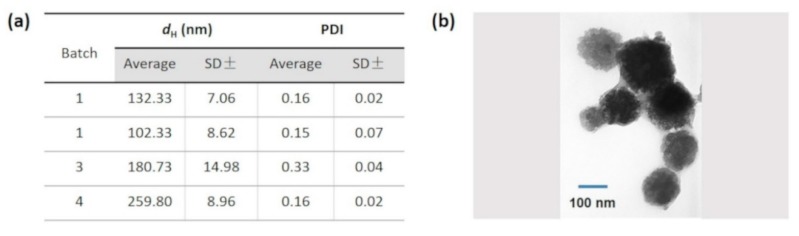
(**a**) Hydrodynamic diameter, (*d*_H_, nm ± SD) and polydispersity index (PDI ± SD) across the different cocaine nanoMIPs batches obtained by DLS analysis; (**b**) TEM image (880,000×) of the cocaine nanoMIPs (batch 1).

Several batches of MIP nanoparticles specific for cocaine, synthesized using the solid phase approach with the same method and monomers composition, were received from the University of Leicester, and these were first characterized by TEM and DLS (Figure 1). As summarized in Figure 1a, DLS analysis showed that the size of the nanoMIPs varied from batch to batch, with an average of the hydrodynamic diameter (*d*_H_) across all the batches of 168.80 ± 68.73 nm. Overall, the one-way ANOVA analysis revealed that the cocaine nanoMIPs *d*_H_ was different across the batches (*F* (3, 74) = 1004.02; *p* < 0.00001). Such a size difference may be caused by the variability occurring during the several manual nanoMIPs syntheses, and this could be easily eliminated by automation of the production process. The TEM analysis, performed on one batch, revealed a small degree of nanoMIPs size heterogeneity within the batch itself (see Figure 1b). According to the TEM analysis, the cocaine nanoMIPs size ranged from 128.98 to 193.09 nm, with an average (±SD) diameter size equal to 148.35 ± 24.69 nm, which is smaller compared to the values achieved by DLS, but in agreement with the size reported in the literature (size range 90–130 nm) [25]. The disparity between the TEM and DLS values might be due to the difference in the samples state during the measurements (dry for TEM; solvated for DLS).

### 3.2. EIS NanoMIPs Sensor Construction

**Figure 2:**
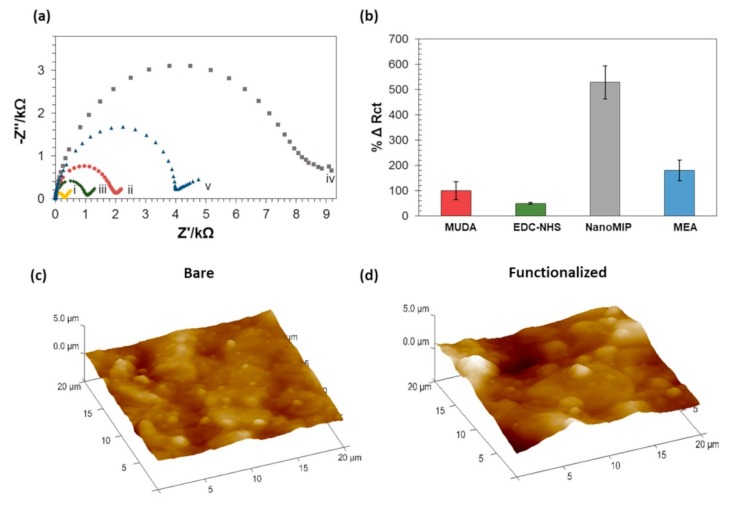
Cocaine nanoMIPs sensor characterization study. (**a**) Nyquist plots for the cocaine nanoMIPs EIS sensor fabrication: (i) bare electrode, (ii) MUDA, (iii) SAM activation via EDC/NHS, (iv) nano-MIP attachment and (v) ethanolamine (MEA) blocking. (**b**) Average of %Rct values (±SD) at each functionalization step obtained by EIS. (**c**,**d**) AFM images of a bare and functionalized electrode surface, respectively.

The nanoMIPs were functionalized with primary amino groups in order to achieve a covalent attachment to the gold sensor surface. Electrodes (DPR C220AT, DropSens, Spain) were cleaned and then functionalized by directly attaching the cocaine nanoMIPs onto the gold sensor chip via amine coupling. The typical EIS spectra are shown in Figure 2a. Briefly, a MUDA SAM was first formed through a thermodynamically favored chemisorption of the thiol groups onto the gold surface [28]. In addition, at a neutral/basic pH, a deprotonation of the MUDA interfacial carboxylic acid groups occurred. Consequently, the electrostatic repulsion between the negatively charged interface and the anionic redox probe induced an increase in Rct value compared to the bare electrode signal [29]. Although there are other thiol compounds that can be used to provide carboxylic groups on gold sensor surface, MUDA was chosen here, as it has been shown previously in our work to be efficient in attaching sensing elements without affecting their binding affinity to the target analytes [30]. After MUDA immobilization on the sensor surface, the carboxylic groups were activated by EDC NHS, which decreased the negative charges of the SAM and, hence, induced a drop of the Rct value [31]. The activated carboxylic groups reacted with the primary amino groups of the cocaine nanoMIPs, thus enabling their covalent attachment. Hence, the Rct value increased due to the nanoMIPs size and insulating properties. Finally, ethanolamine was used to block any unreacted and activated carboxylic groups, thus minimizing the nonspecific binding occurrence. By attaching to the unreacted MUDA, the ethanolamine reduced the negative charges and introduced hydrophilic groups, thus producing a drop of Rct value. Overall the EIS revealed that the nanoMIPs were successfully attached to the electrode surface, although nanoMIPs adsorption onto the surface cannot be completely excluded. Overall, the nanoMIPs immobilization was found to be reproducible, as shown in Figure 2b. Once functionalized, the electrodes were characterized by using AFM analysis, which was conducted on both the bare and the functionalized sensors. Figure 2c,d show the changes in roughness of the surface topography for the bare electrode and for the nanoMIPs functionalized sensor surface, respectively.

### 3.3. EIS NanoMIPs Sensor Optimization

**Figure 3:**
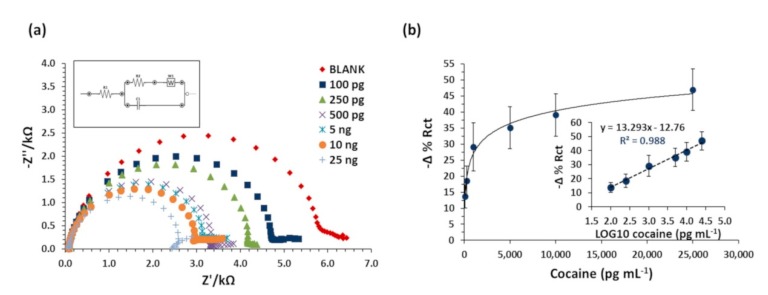
Cocaine cumulative assay performed using the non-optimized cocaine nanoMIPs sensor. (**a**) EIS spectra of blank signal and increasing concentrations of cocaine dissolved in double-distilled water. Inserted graph displays the Randles equivalent circuit used to fit the data. (**b**) The nonlinear and linear calibration (insert graph) curves obtained by plotting the −Δ % Rct values against the cocaine concentration. Error bars refer to the standard deviation of replicates (n = 5).

The sensitivity of the EIS nanoMIPs sensor, fabricated and blocked with ethanolamine (1 M, pH 8.5), was tested by performing a cumulative concentration assay, using cocaine in water (100 pg mL^−1^ to 50 ng mL^−1^). The EIS experimental data (Figure 3a) were fitted in the Randles equivalent circuit, and the Rct values were expressed as Δ % Rct, considering the blank signal value (distilled water) as the starting point. Figure 3b shows the response curve of the sensor when exposed to increasing concentrations of cocaine. The limit of detection (LOD) was calculated as three times the standard deviation of the blank signals and found to be 0.52 ng mL^−1^. No change in Rct values was observed when cocaine was incubated on a control sensor that was functionalized with the same method, but without the nanoMIPs (data not shown).

Nevertheless, to reduce the error bars and enhance the sensor reproducibility, the nanoMIPs sensor was optimized by changing the working solution and optimizing the blocking agents [32,33,34] Therefore, MOPS at pH 7.4 was selected as the working buffer diluent. Then, several blocking reagents were investigated (BSA, milk proteins, Tween 20 and PVA), alone or in combination, as explained above. By comparing the results achieved from these experiments (data not shown), ethanolamine combined with BSA + Tween 20 (pH 7.4) was identified as the optimal blocking condition and was therefore chosen as the best strategy to minimize nonspecific binding and enhance sensor reproducibility.

### 3.4. Sensor Sensitivity and Specificity

**Figure 4:**
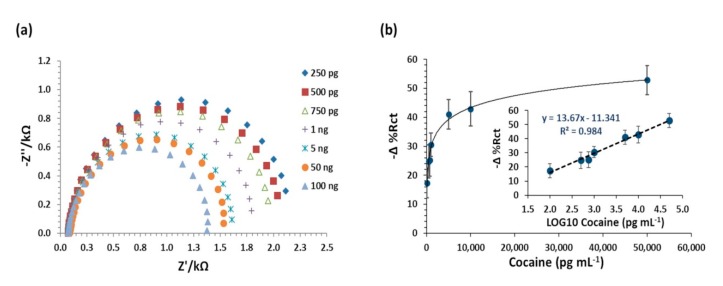
Cocaine cumulative assay performed using the optimized cocaine nanoMIPs sensor. (**a**) Nyquist plot of the data obtained during the cocaine cumulative assay (100 pg mL^−1^ to 50 ng mL^−1^), using the optimized assay condition and fitted by using the simplified Randles equivalent circuit. (**b**) Nonlinear and linear calibration curve of cocaine cumulative assay performed on the optimized cocaine nanoMIPs EIS sensor (DPR C220AT). Error bars refer to the standard deviation of replicates (n = 5).

The cocaine cumulative assay was repeated under the optimized assay and blocking conditions. The experimental data were fitted into the simplified Randles equivalent circuit, and the Rct values were extrapolated with an average of error fitting equal to 2.44% (±1.55%). The Rct values were then expressed as −Δ % Rct and were used to plot the calibration curve. As shown in Figure 4, using these optimized conditions, the sensor was able to detect cocaine at lower concentrations. The R^2^ of the linear calibration curve was equal to 0.984 (*p*-value = 0.00001), and the LOD was found to be 0.24 ng mL^−1^ (0.70 nM).

The EIS nanoMIPs sensor was then tested against morphine, as another commonly trafficked drug of abuse, and levamisole as a common cutting agent [35,36,37,38,39]. Increasing concentrations of each analyte (from 100 pg mL^−1^ to 50 ng mL^−1^) were incubated on the sensor surface, and the EIS spectra were recorded. The data were processed, and the average (±SD) % error of Rct values, as well as the main statistics are reported in Table 1.

**Figure 5:**
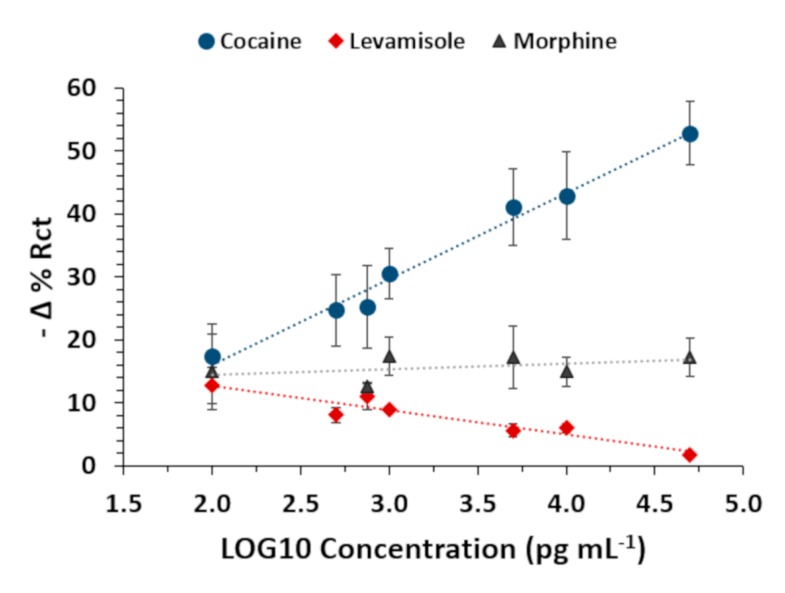
Selectivity study: comparison between cocaine, levamisole and morphine linear calibration curves. Error bars refer to the standard deviation of replicates (n = 5).

The results show that the sensor was not responding to increasing concentrations of morphine (Figure 5). A low response in the opposite direction to that of cocaine was recorded for levamisole. However, although in real drug samples, the cutting agent might be present in lower concentration than cocaine [39,40], in this study, it was tested in a similar concentration range (ng mL^−1^) (i.e., 1.06–1.67 times higher in molarity), thus assessing a worst-case scenario. Furthermore, the specificity of the nanoMIPs used in this work was also tested against paracetamol and caffeine, using a “Pseudo” ELISA assay, and no cross-reactivity was reported [41]. Nevertheless, it should be noted that cutting agents change over time and across different countries, as well as across different regions in the same countries, as reported in retrospective studies [36]. Therefore, the nanoMIPs EIS sensor specificity will need to be reassessed periodically, and further blocking agent optimization studies may be required accordingly.

Overall, the developed EIS nanoMIPs sensor promises to be a valuable alternative to the current onsite screening methods, as a cost-effective, portable, highly sensitive and specific technique. The achieved LOD is slightly above the estimated LOD of the canine olfactory system for cocaine detection [42], but still below the ppb (ng mL^−1^) range required for trace analysis. In addition, the ability of dogs to detect illicit drugs varies according to the breed, training and environmental interference, with the event of cocaine incorrect indication reported to be as high as 26% [4].

Table 2 shows a comparison between the sensor developed in this work with other available analytical tools.

Although mass spectrometry can achieve very low LOD, our EIS nanoMIPs sensor can provide faster results compared to Raman and mass spectroscopy analysis, which require long and tedious sample preparation procedures. The developed sensor can also compete with the extensively used IMS and immunoassays. Indeed, the commercially available IMS has an LOD in the sub ng range (IONSCAN 500DT^®^, Smiths Group plc, London, UK), but it is prone to false-positive results due to the competitive ionization of cutting agents or other environmental compounds [60]. The sensor developed within this work is based on a direct assay format, which decreases the likelihood of false-positive results often linked to the use of competitive inhibition immunoassay screening kits (ELISA kit or lateral flow device) [7,61]. Furthermore, the achieved LOD is well beyond the cocaine cut-off (10–50 ng mL^−1^) of most immuno-based devices in use [9]. Biosensor platforms for drugs of abuse detection have been explored previously, and these have shown low LOD, while being faster and cheaper compared to commercially available analytical tools [62]. As shown in Table 2, most of the developed biosensors are able to detect cocaine in trace concentration and in a wide range of sample matrices (mostly biological samples). Recently, sub µM limit of detection has been achieved by using bioderived [55,56,59] or hybrid nanozyme receptors [53]. However, the nanoMIPs used in this work guarantees higher stability against environmental factors that usually affect bioderived receptors (protein, antibodies and aptamers), such as high or low temperatures and enzymatic degradation. In addition, the sensor response here is due to the detection of direct binding between the nanoMIPs and cocaine at the electrodes interface. On the other hand, aptamers-based sensors mostly rely upon the folding of the aptamers, which occurs when the aptamer binds the target [46], although nonspecific aptamer folding cannot be excluded. Recently, Oliveira and co-workers [52] demonstrated the development of a holographic sensor based on a biomimetic affinity ligand for the detection of cocaine. Although promising preliminary results have been reported, the specificity toward cocaine cutting agents was not assessed.

Compared to the MIP-film-based optical sensors developed by Wren et al. [50] and Nguyen et al. [49], the LOD achieved in our work is far lower (0.24 ng mL^−1^ or 0.70 nM), and the EIS nanoMIPs sensor specificity has been investigated and confirmed against levamisole and morphine. Compared to optical and piezoelectric platforms, the EIS analyzer used in this work is cheaper and easier to miniaturize to obtain a portable device. Furthermore, the faradic EIS technique used here enhances the sensitivity in a sub ppb range, making the developed sensor a promising platform to detect traces of cocaine in environmental samples with high sensitivity and specificity.

## 4. Conclusions

This work has shown that nanoMIPs coupled with an EIS sensor platform can be used to detect cocaine at trace levels. To the best of our knowledge, a sensor based on nanoMIPs and EIS has not been reported previously. The results have shown that cocaine nanoMIPs can be attached onto gold screen-printed electrodes and can be used as a receptor to obtain a highly sensitive EIS sensor for the detection of cocaine (LOD 0.24 ng mL^−1^). The developed sensor has also demonstrated high specificity by being able to discriminate cocaine from morphine and from the most common cocaine cutting agent, namely levamisole. Overall, the nanoMIPs EIS sensor, developed here, can provide onsite, fast and accurate results and, therefore, it can be considered a valid alternative to sniffer dogs or to the current onsite screening methods in use to detect cocaine in environmental samples.

## Figures and Tables

**Table 1:** Statistics and equations of the linear calibration curves achieved during the cumulative assay of the listed analytes.

Analyte	NanoMIPs (Yes/No)	Error % Rct (±SD ^1^)	Linear Equation	R^2^	*p*-Value
Cocaine	Yes	2.44 (±1.55)	y=13.67x − 11.341	0.984	<0.000
Morphine	Yes	3.31 (±1.76)	y =0.8783x + 12.742	0.193	0.353
Levamisole	Yes	1.47 (±1.33)	y = −3.8668x + 20.375	0.879	0.001

^1^ SD = standard deviation; diluent = MOPS pH 7.4.

**Table 2:** Currently available tools and biosensor platforms to detect cocaine in biological and environmental samples and their LOD and linear range.

**Detection Tools Description**	**Sample**	LOD *	**Linear Range**	**Reference**
Dogs Olfactory system	Illicit cocaine vapor	88.3 pM	-	[42]
Transportable Raman spectrometer	Mixture from street sample, seized materials	14.7 mM	-	[43]
Immuno-based kit	Biological and environmental samples	29.4–147 nM	-	[9,44]
Paper spray Ion Trap Mass Spectrometry (IMS)	Surface samples and bulk powder dissolved in liquid	5.9 µM	14.7–588 µM	[45]
	Surface samples	5 ng (sampled area)	-	
Various Hyphenated MS Methods	Liquid sample	5.8 µM	-	[10]
	Outdoor and indoor air	0.07–0.33 ng m^−3^	-	
EIS aptasensor	Wastewater	10 nM	10 nM–50 µM	[46]
	Spiked serum	200 pM	1–8 nM	[47]
Voltammetric aptasensor	Spiked serum	1–8 nM	1–8 nM	[47]
Amperometric immunosensor	Urine Saliva Serum	42.38 nM 10.6 nM 74.16 nM	-	[48]
Potentiometric sensor MIP	Blood serum	-	1 nM–1 mM	[25]
SPR–MIP film	Diluted cocaine	-	0–400 µM	[49]
Optical fiber MIP	Diluted cocaine	-	500–1000 µM	[50]
Electromagnetic piezoelectric acoustic sensor (EMPAS) aptasensor	Diluted cocaine	0.9 µM	2–50 µM	[51]
Holographic sensor—biomimetic receptor	Diluted cocaine	7.1 mM	10–50 mM	[52]
Colorimetric sensor—CTAB-AuNP-L-cyst-ZnSeS QDs hybrid nanozyme	Diluted cocaine	128 nM	20–100 µM	[53]
Voltammetry Sensor—no receptor	Diluted cocaine	10.9 µM	20 µM–100 mM	[54]
Capacitance sensor—Aptamer	Spiked serum Buffer	1.34 pM 7.8 fM	14.5 fM–1.45 pM	[55]
Colorimetric—Au NP Aptamer	Spiked serum	830 pM	2–100 nM (buffer)	[56]
Electrochemical Sensor—β CD	Diluted cocaine in synthetic urine	28.62 nM	25–200 nM	[57]
Electrochemical LFD—β CD Antibody	Synthetic saliva	-	0.03–2.94 µM	[58]
Cyclic Voltammetry—Antibody	water, oral fluids and urine	2.94 fM	2.94 fM–2.9 µM	[59]
EIS-sensor nanoMIPs	Diluted cocaine	0.70 nM	0.30–147 nM	Our work

* When appropriated, the LOD (w/v) was recalculated in molarity (MW = 339.816 g mol^−1^).
